# Clinical Isolates of *Vibrio cholerae* O1 El Tor Ogawa of 2009 from Kolkata, India: Preponderance of SXT Element and Presence of Haitian *ctxB* Variant

**DOI:** 10.1371/journal.pone.0056477

**Published:** 2013-02-19

**Authors:** Braj M. R. N. S. Kutar, Neha Rajpara, Hardik Upadhyay, Thandavarayan Ramamurthy, Ashima K. Bhardwaj

**Affiliations:** 1 Department of Human Health and Diseases, School of Biological Sciences and Biotechnology, Indian Institute of Advanced Research, Koba Institutional Area, Gandhinagar, Gujarat, India; 2 National Institute of Cholera and Enteric Diseases, Beliaghata, Kolkata, India; Charité-University Medicine Berlin, Germany

## Abstract

**Background:**

Increase in the number of multidrug resistant pathogens and the accompanied rise in case fatality rates has hampered the treatment of many infectious diseases including cholera. Unraveling the mechanisms responsible for multidrug resistance in the clinical isolates of *Vibrio cholerae* would help in understanding evolution of these pathogenic bacteria and their epidemic potential. This study was carried out to identify genetic factors responsible for multiple drug resistance in clinical isolates of *Vibrio cholerae* O1, serotype Ogawa, biotype El Tor isolated from the patients admitted to the Infectious Diseases Hospital, Kolkata, India, in 2009.

**Methodology/Principal Findings:**

One hundred and nineteen clinical isolates of *V. cholerae* were analysed for their antibiotic resistance phenotypes. Antibiogram analysis revealed that majority of the isolates showed resistance to co-trimoxazole, nalidixic acid, polymixin B and streptomycin. In PCR, SXT integrase was detected in 117 isolates and its sequence showed 99% identity notably to ICE*Vch*Ind5 from Sevagram, India, ICE*Vch*Ban5 from Bangladesh and VC1786ICE sequence from Haiti outbreak among others. Antibiotic resistance traits corresponding to SXT element were transferred from the parent *Vibrio* isolate to the recipient *E. coli* XL-1 Blue cells during conjugation. Double-mismatch-amplification mutation assay (DMAMA) revealed the presence of Haitian type *ctxB* allele of genotype 7 in 55 isolates and the classical *ctxB* allele of genotype 1 in 59 isolates. Analysis of topoisomerase sequences revealed the presence of mutation Ser83 → Ile in gyrA and Ser85→ Leu in parC. This clearly showed the circulation of SXT-containing *V. cholerae* as causative agent for cholera in Kolkata.

**Conclusions:**

There was predominance of SXT element in these clinical isolates from Kolkata region which also accounted for their antibiotic resistance phenotype typical of this element. DMAMA PCR showed them to be a mixture of isolates with different *ctxB* alleles like classical, El Tor and Haitian variants.

## Introduction

*Vibrio cholerae* is a Gram-negative pathogen that causes cholera, an acute dehydrating diarrhoea which is globally important as it occurs in endemic, epidemic and pandemic forms [1], [2]. *V. cholerae* has been classified on the basis of its somatic O-antigen and more than 200 serogroups have been identified. Out of these, only O1 and O139 are epidemic [1], [2]. The emerging multiple drug resistance in all the bacterial pathogens including *V. cholerae* is complicating the treatment of diseases and therefore is a major public health concern. Chromosome-borne and/or mobile genetic element-borne genes contribute to the drug resistance phenotype of a bacterium. The acquisition and dissemination of antibiotic resistance genes is mediated by mobile genetic elements like plasmids, integrons and transposons [3]. One such transposon is SXT element, an integrative conjugative element (ICE) that integrates and replicates with the host chromosome, can excise itself and be transferred between bacteria by conjugation [4]. ICEs are known to transfer a diverse array of functions including antibiotic resistance genes [4]. SXT element was first reported in 1993 from India; strain O139, MO10 which encoded resistance to sulfamethoxazole, trimethoprim, chloramphenicol and streptomycin [5]. SXT^MO10^-related elements are now present in most *V. cholerae* O139 and O1 clinical isolates [4]–[7]. For evolutionary reasons, *V. cholerae* strains have been continuously changing from classical to El Tor, from O1 to O139, from Ogawa to Inaba, SXT^M010^/R391 hybrids and from plain *ctxB to ctxB* hybrids [6]–[8]. India and Bangladesh have been the haven for evolutionary optimisation of this pathogen and SXT-related ICEs have been characterized from these regions [7], [9]. The ongoing Haiti outbreak has also been predicted to originate from Southeast Asian region [7], [10]–[16] though the controversies still remain regarding the precise geographical source and the etiological agent [15], [16].

In earlier studies from this laboratory, various genetic factors like efflux pumps, plasmids, integrons, *qnr* genes and mutations in topoisomerases were evaluated for their role in conferring antibiotic resistance [17]–[20]. In the present study, *V. cholerae* O1 Ogawa isolated from the patients of Infectious Diseases Hospital (IDH) of Kolkata, India, in 2009, were examined for genetic factors governing their antibiotic resistance profiles. Results revealed the prevalence of SXT element and the absenceof integrons in these isolates. Antibiotic resistance traits and their transferability by conjugation also corroborated the presence of this mobile genetic element. Interestingly, Double-Mismatch-Amplification Mutation Assay (DMAMA) showed the presence of classical, El Tor as well as Haitian *ctxB* variants in these isolates. Mutations in topoisomerase genes *gyrA* and *parC* governed the quinolone resistance phenotype in these isolates.

## Methods

### Bacterial Strains, Genomic and Plasmid DNA Isolation

One hundred and nineteen isolates of *V. cholerae* O1 Ogawa were obtained from patients with acute cholera admitted to the Infectious Diseases Hospital (IDH), Kolkata, India, in 2009 and these patient samples were anonymized. The participants provided their written consent for participating in the study and in case of children, written consent was obtained from their parents. The consent procedure was approved by the Institutional Ethical Clearance Committee of National Institute of Cholera and Enteric Diseases (NICED), Kolkata, from where the samples were obtained for this study. The study was also approved by the Institutional Biosafety Committee (IBSC) of Indian Institute of Advanced Research, Gandhinagar, and the Review Committee on Genetic Manipulation (RCGM) governed by guidelines laid down by Department of Biotechnology, Govt. of India.

*V. cholerae* strains MO10, O1 El Tor N16961, O1 classical Inaba strain 569B were used as controls in various experiments. *Escherichia coli* XL-1 Blue cells were used as recipient in conjugation experiments. Genomic and plasmid DNA isolations were done as described previously [21].

### Antimicrobial Susceptibility Testing

*V. cholerae* isolates were tested for their susceptibility to ampicillin (10 µg), chloramphenicol (30 µg), co-trimoxazole (1.25 µg trimethoprim/23.75 µg sulfamethoxazole), ciprofloxacin (5 µg), gentamicin (10 µg), streptomycin (10 µg), sulfisoxazole (300 µg), trimethoprim (5 µg), tetracycline (30 µg), neomycin (30 µg), nalidixic acid (30 µg), norfloxacin (10 µg), kanamycin (30 µg) and polymixin B (300 units) by the disk diffusion method using commercial disks (HiMedia, Mumbai, India) in accordance with the criteria recommended by Clinical and Laboratory Standards Institute (CLSI) standards [22]. When no interpretive criteria for *V. cholerae* were available based on CLSI guidelines, breakpoints for enterobacteriaceae were applied. *E. coli* ATCC 25922 was used for quality control.

### Polymerase Chain Reactions

Primer pairs L2/L3, qacEΔ1/Sul1B, In-F/In-B were used for detection and characterization of class 1 integrons using the same conditions as described before [17]. Amplification of SXT integrase was carried out using the forward primer 5′- ATGGCGTTATGAGTTAGCTC- 3′ and the reverse primer 5′-GCGAAGATCATGCATAGAC- 3′. For amplification of SXT integrase, conditions used for PCR were similar as described earlier [17] except that annealing was carried out at 57°C for 30 s and extension was carried out at 72°C for 1 min. DMAMA-PCR was carried out to screen for the type of *ctxB* gene present using the primers ctxB-F3, ctxB-F4, Fw-con, Rv-El Tor and Rv-Cla as described recently [23]. PCR amplifications for four topoisomerase genes (*gyrA*, *gyrB*, *parC*, *parE*) were carried out as described earlier [18]. PCR reactions were performed using a PTC-225 DNA Engine Tetrad™ Cycler (MJ Research Inc., MA, USA) and *Pfu* (Fermentas International Inc., Ontario, Canada) or *Taq* DNA polymerases (Bangalore Genei, Bangalore, India).

### Bacterial Conjugation

Two representative SXT-positive isolates (IDH01572 and IDH01738) were tested for their ability to transfer SXT-borne resistance traits to the recipient strain in conjugation experiments according to the published protocols [18]. SXT-negative strain (IDH02095) was included as control in this experiment using the same recipient. The strains IDH01738 (streptomycin resistant), IDH01572 (streptomycin resistant) and IDH02095 (ampicilin and nalidixic acid resistant) were used as donors while *E. coli* XL1-Blue (tetracycline, nalidixic acid resistant and streptomycin sensitive) was used as recipient. Briefly, the recipient and donor strains were mixed in a ratio of 2∶1 on a sterile 0.45 µm nylon membrane (Nytran N, Whatman, PA, USA) and incubated overnight for mating on LB agar at 37°C. The transconjugants were selected on LB agar plates containing appropriate antibiotics. For conjugation between IDH01738, IDH01572 and XL1-Blue, streptomycin (20 µg/mL) and tetracycline (25 µg/mL) were used. For conjugation between IDH02095 and XL1-Blue, selection of ampicillin (25 µg/mL) and tetracycline (25 µg/mL) was used. The transconjugants were confirmed for their authenticity by determination of their antibiotic susceptibility profiles and by PCR for SXT integrase using the donor and recipient strains as controls in both these assays.

### DNA Sequence Analysis

DNA segments amplified from SXT integrase and topoisomerase genes were sequenced and subsequently, the sequences were assembled for each amplicon. The assembled sequences were analysed by nucleotide BLAST search at the National Center for Biotechnology Information (NCBI) website (http://www.ncbi.nlm.nih.gov). Alignment of the topoisomerase sequences used DNASTAR. All new data corresponding to the DNA sequences of SXT integrase and topoisomerase genes were deposited in GenBank and the accession numbers have been mentioned in the appropriate places in results section.

## Results

### Antibiotic Resistance Profiles of Clinical Isolates

**Figure 1:**
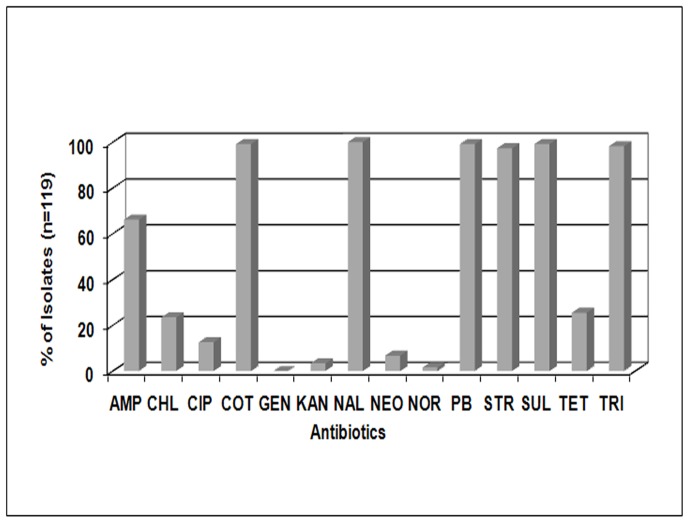
Antibiotic susceptibility profile of 119 clinical isolates from Kolkata, India, in 2009. AMP, Ampicillin; CHL, Chloramphenicol; CIP, Ciprofloxacin; COT, Co-Trimoxazole; GEN, Gentamicin; KAN, Kanamycin; NAL, Nalidixic Acid; NEO, Neomycin; NOR, Norfloxacin; PB, Polymixin B; STR, Streptomycin; SUL, Sulfisoxazole; TET, Tetracycline; TRI, Trimethoprim.

The isolates were identified as *V. cholerae* O1 Ogawa El Tor with standard biochemical tests and serogroup analysis. These isolates showed varying antibiograms but a common resistance profile was clearly evident (Figure 1). The isolates were found to be resistant to nalidixic acid (100%), co-trimoxazole (99.2%), sufisoxazole (99.2%), polymixin B (99.2%), trimethoprim (98.3%) and streptomycin (97.4%). All the isolates showed susceptibility to gentamicin and very few showed resistance to norfloxacin (1.7%) and kanamycin (3.4%). Notably, though all the strains showed resistance to nalidixic acid, resistance to fluorinated quinolones like norfloxacin (1.7%) and ciprofloxacin (12.6%) was not that extensive. Additionally, resistance to SXT-borne traits like chloramphenicol (23.5%) did not correspond to the carriage of other SXT-associated traits like trimethoprim (98.3%), streptomycin (97.4%) and sulfisoxazole (99.2%) indicating the possibility of SXT variants in this population of clinical isolates [6].

### Presence of SXT Element and Class 1 Integron

**Figure 2:**
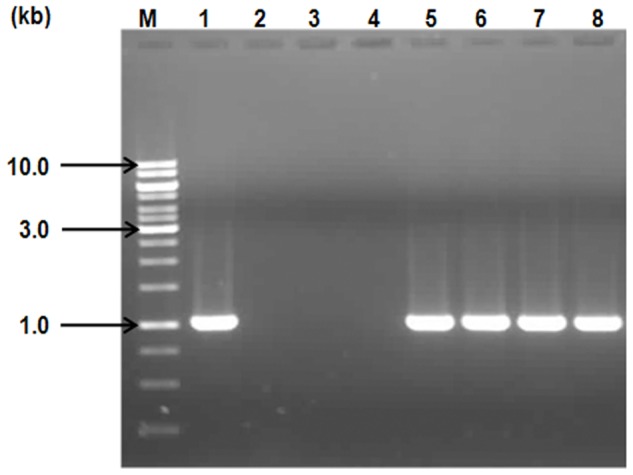
Agarose gel (1%) analysis of PCR product of SXT integrase from IDH isolates and their transconjugants. PCR products obtained using genomic DNA templates from clinical isolates or their transconjugants have been electrophoresed in different lanes as follows: Lane M : 1 kb ladder (Fermentas); Lane 1: Positive control *V.cholerae* O139 MO10; Lane 2: Recipient *E. coli* XL-1 Blue; Lanes 3 and 4: Negative controls of no DNA template and SXT-negative IDH02095 isolate respectively; Lanes 5 and 6 : IDH01572 (SXT-positive) isolate and its transconjugant respectively; Lanes 7 and 8 : IDH01738 (SXT-positive) isolate and its transconjugant respectively.

One hundred and seventeen of the 119 isolates produced a1.0 kb amplicon with primers specific for SXT integrase (Figure 2). Sequence analysis of this amplicon was done using BLAST and sequence for the isolate IDH02596 was deposited into GenBank (**JQ013431**). Results revealed that the SXT sequence had 99% identity to SXT integrase from many other isolates including ICE*Vch*Ind5 from Sevagram, India (**GQ463142**), ICE*Vch*Ban5 from Bangladesh (**GQ463140**) and VC1786ICE sequence from Haiti outbreak (**JN648379**). In all the 117 isolates, presence of SXT element correlated with resistance to co-trimoxazole. All the isolates were negative for class 1 integron in PCR assay.

### Transfer of Resistance Traits by Conjugation

To confirm the transferability of SXT element, conjugation experiments were carried out with two representative SXT-positive isolates (IDH01572 and IDH01738) as donors using *E. coli* XL1-Blue cells as recipients. IDH02095, a SXT-negative isolate, was taken as negative control in this experiment. Transconjugants were obtained with SXT-positive isolates whereas SXT-negative isolates could not yield any transconjugants. PCR and antibiogram analysis of the transconjugants indicated the transfer of SXT element and resistance traits harboured by them (STR, TRI, SUL and COT), from the donor to the recipient cell (Table 1; Figure 2).

**Table 1:** Antimicrobial susceptibility of IDH01572, IDH01738 and their transconjugants.

Isolates	Resistant	Intermediate resistant
IDH01572	AMP, COT, NAL, PB, STR, SUL, TRI	CHL, NEO
IDH01572 transconjugants	CHL, COT, **NAL**, SUL, **TET**, TRI	STR
IDH01738	COT, NAL, PB, STR, SUL, TRI	CIP
IDH01738 transconjugants	COT, **NAL**, SUL, **TET**, TRI	STR

The antibiotic names are as described in legend to figure 1.

Bold face indicates the resistance traits from recipient XL-1Blue cells.

### Presence of Haitian Variant of *ctxB*

DMAMA-PCR was carried out to discriminate the classical, Haitian and El Tor *ctxB* alleles present in these *V. cholerae* isolates as described in a recent report [23]. This assay distinguishes the three *ctxB* alleles based on the mutations specific to each type. Haitian variant carries a mutation at 58^th^ nucleotide corresponding to 20^th^ amino acid (His20 in classical and El Tor → Asn in Haitian allele) which forms the basis of primer ctxB-F3 for Haitian *ctxB* and primer ctxB-F4 for classical *ctxB*. The reverse primer Rv-Cla would anneal to both Haitian as well as classical *ctxB* [23]. El Tor allele would not show amplification in DMAMA-PCR as neither of the forward primers (ctxB-F3 or ctxB-F4) nor the reverse primer (Rv-Cla) would anneal to this *ctxB* variant. PCR results revealed that this population of 119 clinical isolates was a mixture of Haitian (genotype 7) and non-Haitian (genotype 1) classical *ctxB* gene. The Haitian allele was present in 46.2% of the isolates (55 out of 119) that yielded a 191-bp fragment in a PCR with ctxB-F3 and Rv-Cla primers. Rest of the isolates showed either classical *ctxB* allele (59 out of 119) that yielded 191-bp fragment with the primer pair ctxB-F4 and Rv-Cla or El Tor *ctxB* allele (5 out of 119) that did not yield any amplicon in the two PCR assays mentioned above.

### Mutations in Topoisomerases

Out of 119 strains, few representative strains were selected for amplification and sequencing of the Quinolone-Resistance-Determining Regions (QRDRs) from the four topoisomerase genes for GyrA, GyrB, ParC and ParE. Sequences of these genes for the isolate IDH02431were deposited in GenBank. (**JX081540-JX081543**). Results revealed that these isolates carried the mutations encoding Ser83→ Ileu in *gyrA* and Ser85→ Leu in *parC* genes. No mutations were detected in *gyrB* and *parE* genes. The nucleotide BLAST analysis of the sequences from all four topoisomerase genes from Kolkata isolates showed 99% identity with many sequences including the ones from the strains 2010EL-1786 (from Haiti), IEC224 (from Brazil), MJ-1236 (from Matlab, Bangladesh) and M66-2 (from Indonesia).

## Discussion

With the dismal scenario of an alarming increase in the drug resistance of various infectious pathogens, continuous surveillance becomes imperative to understand the pathogens and their changing drug resistance profiles for an effective treatment [3]. The present study was undertaken to determine the drug resistance profiles of one hundred and nineteen clinical isolates of *V. cholerae* O1 El Tor Ogawa from Kolkata, in 2009, and unravel some of the mechanisms that could be responsible for their observed drug resistance phenotypes. Presence of SXT element in majority of the isolates and their resistance to drugs characteristic of SXT element clearly showed the circulation of this genetic factor in the clinical isolates of *V. cholerae*. Sequences of topoisomerases from the representative isolates in this Indian population indicated the presence of a mutation in GyrA (Ser83→ Ileu) and another mutation in ParC (Ser85→ Leu). Though the mutation Ser83→ Ileu in GyrA accounted for the observed nalidixic acid resistance in 100% of the isolates, the effect of the other mutation in ParC could not be explained as the isolates were not resistant to fluoroquinolones like ciprofloxacin and norfloxacin for which this mutation is known to contribute [24], [25]. Resistance profile of these isolates was very similar to the one described earlier for *V. cholerae* strains circulating in India from the year 2004–2007 and also in Haiti [6], [26]. A recent report has described DMAMA-PCR as an effective tool to study the emergence and dissemination of Haitian *ctxB* allele in India [23]. Our study using the DMAMA-PCR also established that the 2009 *V. cholerae* isolates had 46.2% of the Haitian *ctxB* allele corresponding to genotype 7, a new variant of *ctxB* that has been reported from Orissa, India, in 2007 and later in Kolkata from 2006–2011 [6], [23].

Though there are few reports describing different number of *V. cholerae* clinical isolates from India in recent years [6], [7], [23], [25], [27]–[30], our report adds another dimension to these finding from India and abroad to once again show that a large number of the Indian strains as recent as 2009 had similar characteristics of the strain that caused Haiti outbreak. It is established now that UN peacekeepers from Nepal were the actual carriers of the pathogen in Haiti but such isolates have also been circulating in the Southeast Asian regions like India and Bangladesh since last few years [10]–[16], [29], [30]. An earlier report had established the genetic relatedness of the ICE*Vch*HAI 1, an ICE element from the strain that caused Haiti outbreak, with that of ICE*Vch*Ind5, an ICE from an Indian isolate from Sevagram, India, in 1994 [25]. BLAST search of the sequence of SXT integrase in our study showed 99% identity with ICE*Vch*HAI, ICE*Vch*Ind5, ICE*Vfl*Ind1 (from *V. fluvialis*) and ICE*Vch*Ban5; all belonging to the same group 2 of SXT/R391 ICEs. Only 95% identity with ICE*Vch*Ind4 (from O139) with origin in Kolkata (group 1of ICEs) and no relation/identity was observed with the third group of ICEs containing the Matlab variant/ICE*Vch*Ban9 [7], [9], [30]. Persistence of this ICE*Vch*Ind5 element in India till 2005 has also been documented [7]. In this study, the continued presence of ICE*Vch*Ind5 till 2009 and its preponderance in *V. cholerae* O1 El Tor Ogawa clinical isolates is shown from Kolkata. It would be pertinent to state here that in this laboratory, earlier work with *V. fluvialis* clinical isolate BD146 had indicated horizontal transfer of a plasmid between *V. fluvialis* and *V. cholerae* O1 [17]. Results described in this paper again indicate the possibility of transfer of an SXT element between these two *Vibrio* species. Though partially proved, further work would be required to prove the sibling relationships of these ICEs and complete characterization of the genetic content of these ICE elements probed in the present study [7]. These studies aimed at understanding the molecular nature of antibiotic resistance do not really help clinical manangement of the diseases but they are an important insight into the evolution and dissemination of the deadly pathogens that traverse the varied geographical and climatic conditions to give rise to an outbreak or a pandemic.
